# Parasites and competitors suppress bacterial pathogen synergistically due to evolutionary trade‐offs

**DOI:** 10.1111/evo.13143

**Published:** 2016-12-27

**Authors:** Xiaofang Wang, Zhong Wei, Mei Li, Xueqi Wang, Anqi Shan, Xinlan Mei, Alexandre Jousset, Qirong Shen, Yangchun Xu, Ville‐Petri Friman

**Affiliations:** ^1^ Jiangsu Provincial Key Lab for Organic Solid Waste Utilization, National Engineering Research Center for Organic‐based FertilizersNanjing Agricultural UniversityWeigang 1Nanjing210095China; ^2^ Institute for Environmental Biology, Ecology & BiodiversityUtrecht UniversityPadualaan 83584CHUtrechtthe Netherlands; ^3^ Department of BiologyUniversity of YorkWentworth WayYorkYO10 5DDUnited Kingdom

**Keywords:** Experimental evolution, fitness cost, interference competition, phage therapy, *Ralstonia solanacearum*, trade‐off

## Abstract

Parasites and competitors are important for regulating pathogen densities and subsequent disease dynamics. It is, however, unclear to what extent this is driven by ecological and evolutionary processes. Here, we used experimental evolution to study the eco‐evolutionary feedbacks among *Ralstonia solanacearum* bacterial pathogen, *Ralstonia*‐specific phage parasite, and *Bacillus amyloliquefaciens* competitor bacterium in the laboratory and plant rhizosphere. We found that while the phage had a small effect on pathogen densities on its own, it considerably increased the *R. solanacearum* sensitivity to antibiotics produced by *B. amyloliquefaciens*. Instead of density effects, this synergy was due to phage‐driven increase in phage resistance that led to trade‐off with the resistance to *B. amyloliquefaciens* antibiotics. While no evidence was found for pathogen resistance evolution to *B. amyloliquefaciens* antibiotics, the fitness cost of adaptation (reduced growth) was highest when the pathogen had evolved in the presence of both parasite and competitor. Qualitatively similar patterns were found between laboratory and greenhouse experiments even though the evolution of phage resistance was considerably attenuated in the tomato rhizosphere. These results suggest that evolutionary trade‐offs can impose strong constraints on disease dynamics and that combining phages and antibiotic‐producing bacteria could be an efficient way to control agricultural pathogens.

Microbial species interactions can affect disease dynamics by changing the relative and absolute pathogen densities in the host‐associated microbiomes (Mendes et al. 2011; Mueller and Sachs 2015; Wei et al. 2015b; Donaldson et al. 2016). These effects can be driven by ecological dynamics where antagonistic microbes directly reduce pathogen densities via parasitism or competition (Wei et al. 2015a,b), or alternatively, by evolutionary dynamics if pathogen adaptation to phages results in trade‐offs with life‐history traits such as virulence (Friman et al. 2009; Addy et al. 2012a; Bull and Lauring 2014; Friman and Buckling 2014). Despite the recent advances in microbiome research, the relative importance of ecological versus evolutionary dynamics for pathogen density dynamics and disease development are still unclear. Here, we use a direct experimental approach to study the growth dynamics of the plant pathogen *Ralstonia solanacearum*. In presence of a parasitic phage and a bacterial competitor.

Bacteriophages are bacteria‐specific viruses that are bacteriophages are important for regulating bacterial densities across different environments. For example, the densities of the human pathogenic bacterium *Vibrio cholerae* and its parasitic phages show seasonal dynamics where cholera incidences inversely correlate with the phage prevalence in the environment (Faruque et al. 2005). Similarly, phage and bacteria density dynamics have been shown to correlate positively in time on a long‐lived chestnut tree host (Koskella 2013). Phages can regulate bacterial densities and have also been used in applied context of phage therapy to kill the pathogenic bacteria in the site of infection (Levin and Bull 2004). One significant limitation of phage‐mediated control of bacteria is rapid resistance evolution, which can occur in a matter of days in laboratory conditions (Lenski and Levin 1985; Hall et al. 2012; Friman and Buckling 2014; Scanlan et al. 2015). While the evolution of resistance is considerably slower in the natural environments (Gomez and Buckling 2011; Gomez et al. 2015), observations suggest that temporal and spatial patterns can constrain phage‐mediated control of bacterial pathogens in natural communities (Vos et al. 2009; Koskella 2013, 2014). However, evolving resistance does not come for free and often leads to fitness costs in terms of poorer functioning of some other bacterial life‐history trait. Such evolutionary trade‐offs include, for example, reduced bacterial growth (Hall et al. 2012; Scanlan et al. 2015) via loss of phage receptors that are not only used by the phage but also by the bacteria to uptake nutrients (Lenski and Levin 1985), reduced bacterial ability to adapt to abiotic conditions (Scanlan et al. 2015), impairment of mismatch‐repair genes (Pal et al. 2007), and reduced expression of bacterial virulence genes (Addy et al. 2012a). These kind of trade‐offs could expose a chink in the bacterial armor and potentially reduce the survival of phage‐resistant bacteria in complex microbial communities.

Besides parasites, pathogenic bacteria are constantly faced by competition with other bacteria: resource competition can significantly limit the establishment and survival of invading bacterial pathogens in the rhizosphere due to competitive exclusion (Wei et al. 2015b). Bacteria can directly inhibit each other by producing bacteriocins (Ghoul et al. 2015) and antibiotics (Wei et al. 2011; Yuan et al. 2012). While the competitive interactions can have important roles for pathogen densities and disease dynamics (Wei et al. 2011, 2015a), it is possible that pathogens can overcome these constraints via adaptation. For example, bacterial resource consumption patterns can evolve rapidly (Riley et al. 2001; Craig et al. 2005; Gomez et al. 2015) and it is well known that bacteria can evolve resistance to antibiotics (MacLean et al. 2010). Evolving in response to one selection pressure is, however, context dependent and could be altered in the presence of other selective agents (Friman and Buckling 2014; Gomez et al. 2015). Phage‐antibiotic synergy (PAS), where phage selection increases bacterial susceptibility to antibiotics (Chan et al. 2016; Torres‐Barcelo et al. 2016; Torres‐Barcelo and Hochberg 2016), could be an important constraint on pathogen evolution in the presence of both parasites and antibiotic‐producing bacteria. One potential mechanism for PAS is phage‐mediated changes in bacterial cell morphology (cell enlargement, elongation, and filamentation; Zak and Kradolfer 1979), which then affect the rate of phage production and sensitivity to lysis (Comeau et al. 2007; Kamal and Dennis 2015). While PAS has been studied in the context of clinical antibiotics, there are no studies linking PAS with naturally occurring rhizosphere bacteria that produce multiple different antibiotics in agricultural context. Furthermore, it is still somewhat unclear if antibiotic selection makes pathogens more susceptible to phages.

Understanding the interplay between ecological and evolutionary processes for host–pathogen interactions is especially important in the context of plant microbiomes: rhizosphere bacteria compete fiercely for the space and resources with the invading pathogens forming the first line of plant defense. Better understanding of such tripartite interactions could be important from the applied perspective. Microbial‐based biocontrol—the use of naturally occurring competitors, predators, and parasites of the pathogen—has showed promising results for disease control in the plant rhizosphere (Mendes et al. 2011; Mueller and Sachs 2015; Wei et al. 2015b). Biocontrol outcomes are unpredictable for a variety of reasons including temporally changing abiotic conditions (Wei et al. 2011, 2015a) and pathogen resistance evolution to biocontrol agents (Handelsman and Stabb 1996; Duffy et al. 2003).

Here, we used an experimental evolution approach to study how *Ralstonia*‐specific phage parasite and competing *Bacillus amyloliquefaciens* bacterium control *R. solanacearum* pathogen densities alone and in combination in the laboratory in vitro and tomato plant rhizosphere in vivo. *Ralstonia solanacearum* bacterium causes lethal vascular bacterial wilt in many important crops and is spread globally (Hayward 1991). This highly complex pathogen is able to adapt to multiple different environmental niches and isolates vary considerably in their host plant range, geographical distribution, pathogenicity, epidemiological relationships, and physiological properties (Genin and Denny 2012). It has been previously shown that both phages and antibiotic‐producing bacteria can control *R. solanacearum* infection to some extent (Wei et al. 2011, 2015a; Addy et al. 2012a,b; Yamada 2012). However, to our knowledge, there are no studies looking at the interactive effect of phages and antibiotic‐producing bacteria on bacterial wilt dynamics on ecological and evolutionary timescales. *Bacillus amyloliquefaciens* inhibits *R. solanacearum* by producing antibacterial secondary metabolites such as lipopeptides, surfactins, and lantibiotics that target bacterial lipopolysaccharides (Chen et al. 2006, 2007; Yuan et al. 2012), while *Ralstonia*‐specific phages use bacterium as their hosts to produce new phage particles before eventually killing them. We first looked at the effects of the phage and *B. amyloliquefaciens* on pathogen population densities and then quantified the pathogen resistance evolution to both phage and *B. amyloliquefaciens*. These eco‐evolutionary dynamics were further correlated with the host survival in a separate greenhouse experiment. We hypothesized that a phage–*B. amyloliquefaciens* combination should be more effective in controlling pathogen densities compared to phage‐only or *B. amyloliquefaciens*‐only treatments. This effect could be driven by density‐dependent ecological dynamics where higher phage and *B. amyloliquefaciens* densities lead to more efficient pathogen suppression, or alternatively, by evolutionary dynamics where selection leads to evolutionary trade‐offs (Friman and Buckling 2014) or PAS (Comeau et al. 2007; Kamal and Dennis 2015; Torres‐Barcelo et al. 2016).

## Materials and Methods

### STRAINS AND CULTURE CONDITIONS

*Ralstonia solanacearum* strain QL‐Rs1115 (Wei et al. 2011; CGMCC accession No. 9487, China General Microbiology Culture Collection Center) was labeled with red fluorescent protein (abbreviated as QL‐RFP) and grown at 30°C in a liquid nutrient medium (10 g glucose, 5 g tryptone, 3 g beef extract, and 0.5 g yeast extract/l). *Bacillus amyloliquefaciens* strain T‐5 (Tan et al. 2013; CGMCC accession No. 8547, China General Microbiology Culture Collection Center) was labeled with green fluorescent protein (abbreviated as T‐5‐GFP) and grown at 30°C in the liquid nutrient medium (Tan et al. 2015). Fluorescent‐labeled strains were used only in the laboratory experiment, whereas nonlabeled strains were used in the greenhouse experiment. Phage (isolated from Qilin town, Jiangsu province, China, 118°57′ E, 32°03′ N) stock solutions were prepared by coculturing with ancestral QL‐RFP strain for 24 h before chloroforming and centrifuging to purify phages from bacteria. Phage solutions were stored at 4°C (Friman and Buckling 2013). Phage produced clear plaques on bacterial overlays, short tail, and a diameter around 60 nm (Fig. S1). This suggests that it was likely a lytic phage and potentially belonged to the family of *Podoviridae*.

### LABORATORY SELECTION EXPERIMENT IN VITRO AND ISOLATION OF EVOLVED PATHOGEN COLONIES

We first tested the effectiveness of phage and *B. amyloliquefaciens* in vitro by using factorial experimental design with four treatments where *R. solanacearum* bacteria were cultured alone (control treatment), in the presence of *B. amyloliquefaciens* T‐5‐GFP (competition treatment), phage (phage treatment), or both phage and *B. amyloliquefaciens* T‐5‐GFP (combinatory treatment). We performed the experiment on a 48‐well microplate with 700 μl volumes per population and used only the 24 wells in the middle of the microplate for our experimental populations (the edge wells were filled with dH2O to prevent evaporation). Each microcosm was initially inoculated with approximately 10^6^ *R. solanacearum* cells. Six of the microcosms were inoculated with approximately 10^5^ cells of *B. amyloliquefaciens* T‐5‐GFP bacterium, six microcosms with approximately 10^6^ phage particles, and six microcosms with approximately 10^5^ cells of *B. amyloliquefaciens* T‐5‐GFP and 10^6^ phage particles. The remaining six microcosms contained only the pathogen. The microplate was incubated at 30°C with shaking (170 rpm), and at every 24 h, we measured the growth of bacteria with SpectraMax M5 Plate reader (Molecular Devices, Sunnyvale, CA) by characterizing the average fluorescence intensity. *Ralstonia solanacearum* growth was measured as a red fluorescence signal (excitation: 587 nm, emission: 610 nm) and *B. amyloliquefaciens* T‐5‐GFP growth as a green fluorescence signal (excitation: 485 nm, emission: 533 nm). Subsamples of all populations (100 μl) were cryopreserved at –80°C in 15% glycerol at 48‐ and 96‐h sampling points to further study the evolutionary dynamics. To this end, we isolated both bacteria and phages from three randomly selected replicate populations from each treatment at both time points (bacteria from every treatment and phages from phage‐containing treatments). Bacteria were isolated by serial dilution and plating on semiselective medium (M‐SMSA) (Elphinstone et al. 1996). After 48 h of growth at 30°C, 24 colonies were picked randomly from each treatment replicate population, grown individually for 24 h at 30°C in liquid medium in 96‐well microplates and cryopreserved at –80°C in 15% glycerol. Similarly, phage populations were isolated from the same corresponding populations where the bacteria were isolated with chloroform treatment and stored at 4°C for evolutionary measurements (Friman and Buckling 2014).

### MEASURING EVOLUTIONARY CHANGES IN PATHOGEN RESISTANCE TO PHAGE AND PHAGE INFECTIVITY

Pathogen resistance and phage infectivity were assayed at the population level as the proportion of resistant bacteria by using spotting assays (Scanlan et al. 2015). Briefly, overnight‐grown bacterial colonies were mixed with cooled‐down soft agar (with 7% agar concentration) and 2 μl of the associated phage population was spotted over the bacterial culture. Bacterial resistance and phage infectivity were determined after 24‐h growth as the proportion of bacterial lawns that had plaques, which is indicative of bacterial lysis due to phage infection. To study potential coevolutionary changes, the resistance of bacteria isolated at 48‐h time point was tested against phages isolated from the past (0 h), contemporary (48 h), and future (96 h) time points. Similarly, the infectivity of phage isolated at 48‐h time point was tested against bacteria isolated from the past (0 h), contemporary (48 h), and future (96 h) time points.

### MEASURING EVOLUTIONARY CHANGES IN PATHOGEN DEFENSE AGAINST ANTIBIOTICS PRODUCED BY *B. amyloliquefaciens* T‐5‐GFP

To reveal potential trade‐offs, the same *R. solanacearum* colonies that were used for phage resistance assays were used to determine the evolution of pathogen defense against antibiotics produced by ancestral *B. amyloliquefaciens* T‐5‐GFP strain. Instead of testing any specific antibiotic, we tested the inhibitory potential of all potential exoenzymes produced by the ancestral *B. amyloliquefaciens* T‐5‐GFP strain. Briefly, the *B. amyloliquefaciens* T‐5‐GFP was cultured in the liquid nutrient medium for 36 h, after the bacterial suspension was centrifuged at 10,000 × *g* for 10 min and filtered through a 0.22 μm syringe filter to remove bacteria. Evolved and ancestral *R. solanacearum* colonies were then grown at 30°C (200 μl of liquid nutrient medium) in 96‐well microplates in the absence (5 μl of media added) or presence of *B. amyloliquefaciens* T‐5‐GFP supernatant (5 μl of supernatant added). Inhibition was measured as the difference of bacterial growth in the absence (OD_600m_) and presence (OD_600t_) of supernatant as follows: Inhibition effect = (OD_600m_ – OD_600t_)/OD_600m_ × 100%.

### MEASURING EVOLUTIONARY CHANGES IN BACTERIAL FITNESS COST (GROWTH IN THE ABSENCE OF PHAGE AND COMPETITOR)

To test whether bacterial evolution incurred fitness costs, the growth of evolved bacteria (isolated at 96‐h sampling point) was measured in the absence of phages and *B. amyloliquefaciens* T‐5‐GFP in 96‐well microplates (at 30°C in 200 μl of nutrient broth) as optical density (600 nm at every 4 h for 24 h). These data were used to count bacterial maximum density (growth at 24‐h time point) and maximum growth rate per hour.

### THE EFFECT OF COMPETITORS AND PARASITES ON BACTERIAL WILT DISEASE DEVELOPMENT, PATHOGEN DENSITIES, AND PATHOGEN EVOLUTION IN THE TOMATO RHIZOSPHERE

Tomato seeds (cultivar Hezuo 903) were surface‐sterilized by immersing them in 70% alcohol for 1 min. Seeds were then washed with sterilized water, immersed in household bleach (2% NaClO) for 5 min, and finally rinsed six times with sterile water. Seeds were germinated in darkness at 25°C on water‐wetted filter paper in a Petri dish. Germinated seeds were aseptically transferred to eight‐plant seeding tray inserts containing 10 kg of autoclaved soil (at 121°C for 2 h) collected from Qilin Town, Nanjing (Wei et al. 2011). A total of 48 plants were used for each treatment (12 plants per replicate, *N* = 3). The greenhouse experiment included the same four treatments as described for in vitro tests. Seedlings were grown in a controlled growth chamber at 28–35°C under a 16‐h light and 8‐h dark cycle. At three‐leaf stage, a cell suspension of *B. amyloliquefaciens* T‐5 bacterium was inoculated into the soil of the seedlings with a final density of approximately 10^8^ cells per gram of dry weight soil. The cell suspension of *R. solanacearum* strain QL‐Rs1115 (10^6^ cells per gram dry weight soil) was inoculated 2 days later. Five days after the inoculation of *R. solanacearum*, the suspensions of phage particles were added with a final density of approximately 10^7^ phage particles per gram of dry weight soil. Plants were then grown in greenhouse conditions for 70 days with natural temperature and light variation and watered regularly with sterilized water. Bacterial wilt disease progression was determined at the end of the experiment 40 days after pathogen inoculation. Disease index was recorded based on a scale ranging from 0 to 4 (Tans‐Kersten et al. 2004), where 0 denotes zero disease incidence, 1 denotes 1–25% disease incidence, 2 denotes 26–50% disease incidence, 3 denotes 51–75% disease incidence, and 4 denotes 76–100% disease incidence.

At the end of the experiment, we randomly chose three healthy plants and measured *R. solanacearum* population densities in the rhizosphere soil. To this end, whole plants were removed, excess soil removed gently by shaking and remaining rhizosphere soil mixed with sterile water and serially diluted and plated on M‐SMSA selective medium. After incubation for 2 days at 30°C, *R. solanacearum* colonies were counted per gram dry weight of soil and 24 colonies per replicate plant in each treatment were isolated to determine *R. solanacearum* resistance evolution to ancestral *B. amyloliquefaciens* T‐5 competitor as described above. Phage resistance to ancestral phage was measured as the *R. solanacearum* density reduction in liquid nutrient broth media. Briefly, *R. solanacearum* colonies were grown in the presence (5 μl of media containing 10^8^ phage particles) and the absence (5 μl of media) of ancestral phage at 30°C in 200 μl of nutrient broth media for 24 h. Resistance to phage was measured as the difference of bacterial growth in the absence (OD_600a_) and presence (OD_600p_) of phage as follows: Inhibition by the phage = (OD_600a_ – OD_600p_)/OD_600a_ × 100%.

### STATISTICAL ANALYSIS

All time‐dependent data wer analyzed with repeated‐measures ANOVA by using time and factors as explanatory variables. Factorial two‐way ANOVA was used for all other analyses. When needed, data were square‐root or log10‐transformed before the analyses in order to meet the parametric model assumptions (normality and homogeneity of variance). The rate of *R. solanacearum* inhibition was calculated as the difference in *R. solanacearum* growth alone versus in the presence of phage or *B. amyloliquefaciens*. Bacterial resistance to phages was determined as the proportion of resistant colonies per treatment replicate, and phage infectivity as the proportion of susceptible, *R. solanacearum* colonies per treatment replicate. All proportional data were arcsine‐transformed before the analyses to meet the parametric model assumptions. Disease index was calculated as described by: Disease index = [∑ (The number of diseased plants in the disease index category × disease index category)/ (Total number of plants used in the experiment × highest disease index category)] × 100%. All analyses were conducted with R (R Core Team 2014).

## Results

### PHAGE‐ and *B. amyloliquefaciens*‐MEDIATED CONTROL OF PATHOGEN POPULATION DENSITIES IN THE LABORATORY EXPERIMENT

**Figure 1:**
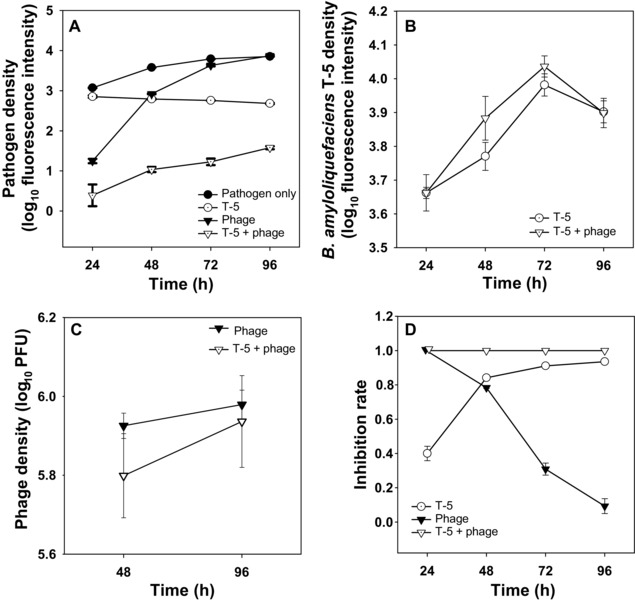
The microbial population dynamics during the short‐term laboratory experiment. The population densities of *Ralstonia solanacearum* pathogen QL‐RFP (A), *B. amyloliquefaciens* T‐5‐GFP competitor (B), phage (plaque forming units, i.e. PFU) (C) and the pathogen inhibition rates in different treatments (D). All bars show ± 1 SE.

Phage negatively affected on the pathogen density at the beginning of the experiment, but this effect vanished through time (Fig. 1A, D; Table 1a, d). *Bacillus amyloliquefaciens* had an even stronger suppressive effect (Fig. 1A, D; Table 1a, d), and even though some changes in time were observed, the reduction was more consistent compared to the effect of the phage (Fig. 1A, D; Table 1a, d). Interestingly, phage and *B. amyloliquefaciens* decreased pathogen densities most efficiently in combination indicative of a synergistic effect (Fig. 1A, D; Table 1a, d). One explanation for this could be that both phages and *B. amyloliquefaciens* had higher population densities when cultured together leading to more efficient pathogen density reduction. However, no difference was found between *B. amyloliquefaciens* densities in single versus combinatory treatments (Fig. 1B, Table 1b), and similarly, *B. amyloliquefaciens* had only a very small negative effect on the phage densities (Fig. 1C, Table 1c). These results suggest that phage and *B. amyloliquefaciens* showed additive synergistic effects at reducing pathogen densities.

**Table 1:** Statistical analyses

Source of variation	df	sum of squares within groups	mean square within groups	*F*	*P*
(a) The *Ralstonia solanacearum* pathogen population densities (laboratory experiment)					
Error: factor(id)					
Phage	1	33.66	33.66	5235	<2 × 10^–16***^
T5	1	42.58	42.58	6621	<2 × 10^–16***^
Phage × T5	1	6.73	6.73	1047	<2 × 10^–16***^
Residuals	20	0.13	0.01		
Total	23	83.1	82.98		
Error: Within					
Time	3	17.359	5.786	1057.37	<2 × 10^–16***^
Phage × Time	3	8.691	2.897	529.38	<2 × 10^–16***^
T5 × Time	3	5.664	1.888	345.02	<2 × 10^–16***^
Phage × T5 × Time	3	0.418	0.139	25.48	9.37 × 10^–16***^
Residuals	60	0.328	0.005		
Total	72	32.46	10.715		
(b) The *B. amyloliquefaciens* T‐5 competitor population densities (laboratory experiment)					
Error: factor(id)					
Phage	1	0.02024	0.020238	4.904	0.0512
Residuals	10	0.04127	0.004127		
Total	11	0.06151	0.024365		
Error: Within					
Time	3	0.7632	0.25441	262.11	<2 × 10^–16***^
Phage × Time	3	0.0269	0.00895	9.226	0.000177^***^
Residuals	30	0.0291	0.00097		
Total	36	0.8192	0.26433		
(c) The phage population densities (laboratory experiment)					
Error: factor(id)					
T5	1	0.02159	0.02159	8.511	0.0434*
Residuals	4	0.01015	0.002537		
Total	5	0.03174	0.024127		
Error: Within					
Time	1	0.0274	0.027399	2.464	0.192
T5 × Time	1	0.00524	0.005242	0.471	0.53
Residuals	4	0.04448	0.011119		
Total	6	0.07712	0.04376		
(d) The pathogen inhibition rates by *B. amyloliquefaciens* T‐5 competitor in different treatments (laboratory experiment)					
Error: factor(id)					
Phage	1	0.0113	0.0113	8.288	0.0115*
T5	1	1.2083	1.2083	889.867	9.04 × 10^–15***^
Residuals	15	0.0204	0.0014		
Total	17	1.24	1.221		
Error: Within					
Time	3	0.313	0.1043	129.3	<2 × 10^–16***^
Phage × Time	3	1.0699	0.3566	441.9	<2 × 10^–16***^
T5 × Time	3	0.9298	0.3099	384.1	<2 × 10^–16***^
Residuals	45	0.0363	0.0008		
Total	54	2.349	0.7716		
(e) The evolution of pathogen resistance to ancestral phage (laboratory experiment)					
Error: factor(id)					
Phage	1	8.435	8.435	191.7	7.16 × 10^–16***^
T5	1	0.044	0.044	1.01	0.344
Phage × T5	1	0.031	0.031	0.71	0.424
Residuals	8	0.352	0.044		
Total	11	8.862	8.554		
Error: Within					
Time	1	0.02193	0.021934	2.399	0.16
Phage × Time	1	0.00638	0.00638	0.698	0.4276
T5 × Time	1	0.02147	0.02147	2.348	0.1639
Phage × T5 × Time	1	0.03263	0.03263	3.57	0.0955
Residuals	8	0.07313	0.00914		
Total	12	0.15554	0.091554		
(f) The evolution of pathogen resistance to contemporary phages (laboratory experiment)					
Error: factor(id)					
T5	1	0.09891	0.09891	7.412	0.0529
Residuals	4	0.05338	0.01334		
Total	5	0.15229	0.11225		
Error: Within					
Bacterial isolation time point	2	5.535	2.7674	342.3	1.78 × 10^–08***^
T5 × Bacterial isolation time point	2	0.28	0.1399	17.3	0.00124^**^
Residuals	8	0.065	0.0081		
Total	12	5.88	2.9154		
(g) The phage infectivity to contemporary pathogen (laboratory experiment)					
Error: factor(id)					
T5	1	0.0497	0.0497	1.254	0.326
Residuals	4	0.1586	0.03964		
Total	5	0.2083	0.08934		
Error: Within					
Phage isolation time point	2	4.644	2.3221	105.205	1.8 × 10^–06^***
T5 × Phage isolation time point	2	0.035	0.0176	0.797	0.484
Residuals	8	0.177	0.0221		
Total	12	4.856	2.3618		
(h) The susceptibility of evolved pathogen to antibiotics produced by ancestral T‐5 *B. amyloliquefaciens* competitor					
(laboratory experiment)					
Error: factor(id)					
Phage	1	0.10846	0.10846	107.217	6.54 × 10^–06***^
T5	1	0.01482	0.01482	14.652	0.00503**
Phage × T5	1	0.10846	0.00267	2.635	0.14319
Residuals	8	0.00809	0.00101		
Total	11	0.23983	0.12696		
Error: Within					
Time	1	0.026521	0.026521	18.234	0.00272**
Phage × Time	1	0.011865	0.011865	8.157	0.02128*
T5 × Time	1	0.005042	0.005042	3.466	0.09966
Phage × T5 × Time	1	0.016804	0.016804	11.553	0.00937**
Residuals	8	0.011636	0.001454		
Total	12	0.071868	0.061686		
(i) The evolved pathogen maximum density in the absence of phage or competitor (laboratory experiment)					
Phage	1	0.163	0.163	24.565	0.001
T5	1	0.252	0.252	38.081	<0.001
Phage × T5	1	0.145	0.145	21.833	0.002
Residual	8	0.053	0.00663		
Total	11	0.613	0.0557		
(j) The evolved pathogen maximum growth rate in the absence of phage or competitor (laboratory experiment)					
Phage	1	0.0000553	0.0000553	0.674	0.435
T5	1	0.000108	0.000108	1.321	0.284
Phage × T5	1	0.0000059	0.0000059	0.0719	0.795
Residual	8	0.000656	0.0000821		
Total	11	0.000826	0.0000751		
(k) The tomato wilt disease incidence (greenhouse experiment)					
Phage	1	102.083	102.083	10.419	0.012
T5	1	198.047	198.047	20.213	0.002
Phage × T5	1	0	0	0	1
Residual	8	78.385	9.798		
Total	11	378.516	34.411		
(l) The pathogen density in the rhizosphere soil (greenhouse experiment)					
Phage	1	0.322	0.322	22.706	<0.001
T5	1	5.798	5.798	409.467	<0.001
Phage × T5	1	0.32	0.32	22.586	<0.001
Residual	12	0.17	0.0142		
Total	15	6.609	0.441		
(m) The evolution of pathogen resistance to ancestral phage (greenhouse experiment)					
Phage	1	6.29 × 10^–07^	6.29 × 10^–07^	0.327	0.583
T5	1	0.0000162	0.0000162	8.425	0.02
Phage × T5	1	2.32 × 10^–06^	2.32 × 10^–06^	1.201	0.305
Residual	8	0.0000154	1.93 × 10^–06^		
Total	11	0.0000346	3.15 × 10^–06^		
(n) The evolution of pathogen resistance to antibiotics produced by ancestral T‐5 *B. amyloliquefaciens* competitor					
(greenhouse experiment)					
Phage	1	0.0234	0.0234	138.235	<0.001
T5	1	0.00629	0.00629	37.235	<0.001
Phage × T5	1	0.00216	0.00216	12.807	0.007
Residual	8	0.00135	0.000169		
Total	11	0.0332	0.00302		

### BACTERIA–PHAGE COEVOLUTION DURING THE LABORATORY EXPERIMENT

**Figure 2:**
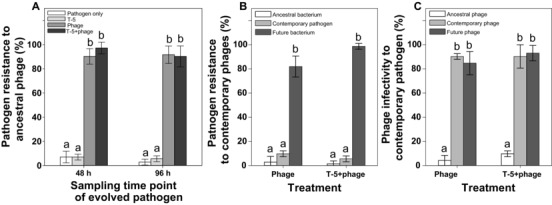
Pathogen–phage coevolutionary dynamics during the short‐term laboratory experiment. (A) The proportion of evolved pathogen (QL‐RFP) that is resistant to ancestral phage in pathogen‐only, competitor, phage, and combinatory treatments. (B) The proportion of resistant pathogen isolated from the past, contemporary, and future time points to contemporary phage populations. (C) The proportion of infected pathogens isolated from the contemporary time point to phage populations isolated from the past, contemporary, and future time points. All bars show ± 1 SE, different letters denote pairwise difference between the treatments test. Tukey? (*P* < 0.05).

Phage selection led to evolution of high levels of phage resistance both in the absence and presence of *B. amyloliquefaciens*, while no increase in resistance was observed when pathogen had evolved alone or in the competition treatment (Fig. 2A, Table 1e). The sampling time point (after 48 or 96 h of selection) did not affect the level of phage resistance to ancestral phage (Fig. 2A, Table 1e).

To explore changes in bacteria–phage coevolution, we measured the resistance of pathogen isolated from the past (0 h), contemporary (48 h), and future (96 h) time points to phages isolated at the contemporary time point (48 h, Fig. 2B). Similarly, we measured the contemporary phages’ (48 h) ability to infect pathogen isolated from the past (0 h), contemporary (48 h), and future time points (96 h, Fig. 2C). We found that pathogen and phage underwent short‐term arms race coevolutionary dynamics in both the absence and presence of competitor: both the bacterial resistance and phage infectivity increased in time (Fig. 2B, C; Table 1f–g). In other words, pathogens from the future were more resistant to the contemporary phages compared to the pathogens from the past, while phages from the future were more infective to the contemporary pathogen compared to the phages from the past. Bacteria–phage coevolution was not affected by the presence of *B. amyloliquefaciens* competitor (Fig. 2B, C; Table 1f–g). These results suggest that *R. solanacearum* pathogen and the phage coevolved rapidly in the laboratory experiment leading to increase in bacterial resistance and phage infectivity.

### EVOLUTION OF BACTERIAL RESISTANCE TO *B. amyloliquefaciens* T‐5‐GFP COMPETITOR

**Figure 3:**
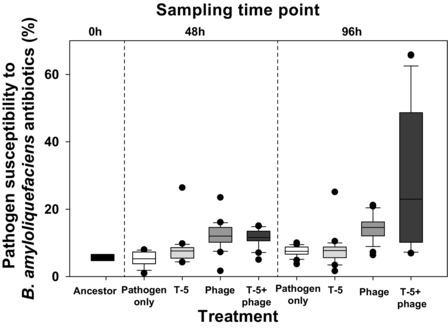
The evolved pathogen susceptibility to antibiotics produced by ancestral *B. amyloliquefaciens* T‐5 strain during the short‐term laboratory experiment. Panels show the susceptibility of *R. solanacearum* pathogen to the antibiotics produced by ancestral *B. amyloliquefaciens* T‐5 strain at 0‐h (ancestor), 48‐h, and 96‐h time points. All bars show SEM ± 1.

We found that exposing pathogen to phage affected its ability to resist antibiotics produced by *B. amyloliquefaciens* (Fig. 3, Table 1h). While the ancestral and evolved pathogens from the control and competitor treatments showed very low and similar susceptibilities to *B. amyloliquefaciens* antibiotics, evolved pathogens isolated from the phage‐only and the phage–competitor treatments showed increased susceptibility to *B. amyloliquefaciens* antibiotics compared to control treatments (Fig. 3, Table 1h). Crucially, the pathogen susceptibility to *B. amyloliquefaciens* antibiotics increased significantly the longer the bacteria had been coevolving with the phage and became greatest in the combinatory treatment (Fig. 3, Table 1h). These results suggest that evolving resistance to phage leads to increased susceptibility to antibiotics produced by *B. amyloliquefaciens* T‐5 strain.

### FITNESS COST OF BACTERIAL ADAPTATION DURING THE LABORATORY EXPERIMENT

**Figure 4:**
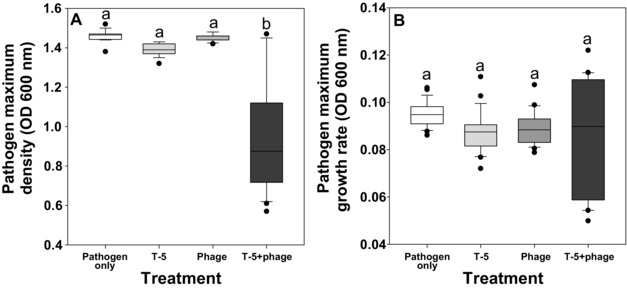
The cost of adaptation during the short‐term laboratory experiment (the relative growth of evolved pathogen in monocultures). The growth of evolved pathogens (isolated at 96‐h time point) in the absence of enemies in pathogen‐only, competitor, phage, and combinatory treatments. (A) Pathogen maximum density, (B) pathogen maximum growth rate. In all panels, all bars show SEM ± 1 and different letters denote pairwise difference between different treatment (*P* < 0.05).

To determine the costs associated with evolving with phages and competitors, we measured both the maximum densities and maximum growth rates of evolved pathogen populations in the absence of phage and competitor. While phage and T‐5 selection alone led to reduced pathogen maximum densities, this was driven by synergistic interaction and clear reduction in pathogen maximum densities was observed only in the combinatory treatment (Fig. 4A, Table 1i). Phage and *B. amyloliquefaciens* T‐5 selection had no effect on the pathogen growth rate (Fig. 4B, Table 1j). These results suggest that *R. solanacearum* pathogen suffered a relatively higher cost of adaptation in the combinatory treatment.

### THE EFFECT OF COMPETITORS AND PARASITES ON PATHOGEN DENSITIES, RESISTANCE EVOLUTION, AND BACTERIAL WILT DISEASE DEVELOPMENT IN THE TOMATO RHIZOSPHERE

**Figure 5:**
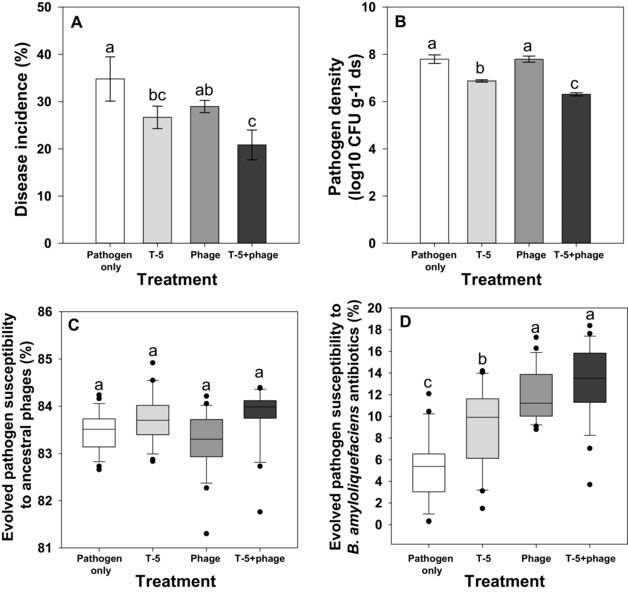
Bacterial wilt disease incidence and pathogen density and evolutionary dynamics during the greenhouse experiment. Panels show bacterial wilt disease incidence (A), pathogen densities in the rhizosphere (B), pathogen resistance evolution to ancestral phages (C), and pathogen susceptibility to antibiotics produced by ancestral *B. amyloliquefaciens* T‐5 strain (D). All bars show SEM ± 1 and different letters denote pairwise difference between different treatment (*P* < 0.05).

Phage and competitor reduced disease incidence (the proportion of wilted plants showing disease symptoms) when applied alone (Fig. 5A, Table 1k). However, the effect of competitor was relatively stronger showing similar synergy with the phage as observed in the laboratory experiment (Fig. 5A, Table 1k; despite the nonsignificant interactions significant contrasts found with one‐way ANOVA). Pathogen densities generally increased from the initial inoculation of 10^6^ CFU/g soil reaching levels comparable to field conditions (Wei et al. 2011, 2015b). Crucially, the pathogen densities correlated well with the level of disease incidence: even though phage and competitor reduced pathogen densities alone, they had the strongest effect when applied together (Fig. 5B, Table 1l). To examine evolutionary changes in the rhizosphere, we compared the resistance of evolved pathogens in the end of the greenhouse experiment. Only low levels of resistance were observed in general and pathogens showed clearly reduced growth in the presence of the ancestral phage regardless of their evolutionary history (Fig. 5C). Interestingly, pathogens that had evolved in the presence of competitor showed slightly higher susceptibility to the ancestral phage compared to other treatments (Fig. 5C, Table 1m). Despite the low levels of resistance, pathogens isolated from the phage treatments showed increased susceptibility to the antibiotics produced by ancestral *B. amyloliquefaciens* T‐5 strain (Fig. 5D, Table 1n). Together, these results demonstrate that phages and competitors can suppress bacterial pathogen synergistically due to evolutionary trade‐off and that in vitro experiments can qualitatively predict microbial interactions in the rhizosphere in vivo.

## Discussion

Here, we studied how *B. amyloliquefaciens* bacterial competitor and parasitic phage affected the population densities and resistance evolution of a *R. solanacearum* pathogen alone and in combination in the laboratory and plant rhizosphere. We found that *B. amyloliquefaciens* exerted stronger antagonism toward the pathogen compared to the phage. In addition, the combination of phage and *B. amyloliquefaciens* showed additive synergistic effects and reduced pathogen densities and bacterial wilt disease incidence most effectively. Rather than density effects, this synergy could be explained with phage‐driven increase in phage resistance, which made pathogens more susceptible to antibiotics produced by *B. amyloliquefaciens*. Such evolutionary trade‐offs could offer novel ways to control bacterial infections both in medical and agricultural settings.

We found that pathogen was able to evolve resistance to phage very rapidly under laboratory conditions, which also likely explains the loss of phage‐mediated pathogen control. However, the phages were also able to coevolve and become more infective, which raises a question: why was the phage unable to evolve to control pathogen population densities? One explanation for this is that our sampling regime was not frequent enough to capture phage‐mediated reduction in pathogen densities (Friman and Buckling 2013, 2014). Moreover, evolving resistance to a phage incurred only a weak cost (slightly reduced growth rate), which likely weakened the phage‐mediated density regulation allowing rapid fixation of new resistance mutations in the pathogen populations. It is also possible that evolving a higher infectivity was costly for the phage leading to less effective reproduction. Even though more detailed studies are needed to uncover the exact mechanism, our results are in line with previous studies reporting continuous phage–bacteria coevolutionary arms race even in the absence of bacterial population density regulation by the phage (Hall et al. 2011). In contrast, we found that *B. amyloliquefaciens* reduced pathogen densities effectively and consistently in the laboratory conditions, which is in line with previous findings (Tan et al. 2013). No evidence for pathogen resistance evolution to *B. amyloliquefaciens* was found. One potential explanation is that the timescale of the laboratory experiment was not long enough for the emergence of resistance mutations. Moreover, *B. amyloliquefaciens* is known to simultaneously produce many different lipopeptide antibiotics (Ongena and Jacques 2008). Even though we did not characterize the antibiotic composition in more detail, it has been shown that concurrent selection by multiple clinical antibiotics is less likely to select for increased resistance (Davies and Davies 2010), which could have also explained the lack of resistance evolution in our experiment.

Interestingly, phage and *B. amyloliquefaciens* had additive synergistic effects leading to highest reduction in pathogen densities. This result could be explained by ecological and/or evolutionary dynamics. First, it is possible that phage and *B. amyloliquefaciens* had higher densities when cultured together, which could have led to more efficient pathogen density reduction. In contrast, we found that phage and *B. amyloliquefaciens* had equally high, or even slightly lower, densities when cultured together versus alone. This suggests that the synergistic antagonism was less likely to be explained by population density effects. We next looked if phage–*B. amyloliquefaciens* synergy could be explained by evolutionary constraints. In support for this, we found that phage selection increased pathogen susceptibility to the antibiotics produced by *B. amyloliquefaciens* T‐5‐GFP ancestral bacteria. Phages often target the receptors in LPS (lipopolysaccharides) (Tamaki et al. 1971; da Costa et al. 2014), which is also the target of *B. amyloliquefaciens* produced lipopeptides (Sequeira and Graham 1977). Therefore, it is possible that phage‐mediated changes in *R. solanacearum* LPS also affected its susceptibility to *B. amyloliquefaciens* antibiotics. This result is line with a recent finding where phage selection was shown to impair the outer membrane porin M (OprM) of the multidrug efflux systems MexAB and MexXY of human opportunistic pathogen *Pseudomonas aeruginosa* (Chan et al. 2016). Together, these results suggest that PAS could efficiently control bacterial infections in both agricultural and clinical contexts. We also found that evolving in the presence of both phage and *B. amyloliquefaciens* incurred a relatively higher fitness cost in terms of reduced pathogen growth in the absence of phage or competitor (maximum density). This growth cost could also have resulted from defective LPS and could have made the pathogen less infective.

The population and evolutionary dynamics showed qualitatively similar patterns between laboratory and greenhouse experiments. First, the pathogen density dynamics correlated well with bacterial wilt disease dynamics in the rhizosphere and even though phage alone had no clear effect on disease incidence or pathogen densities, *B. amyloliquefaciens* reduced pathogen densities both in the absence and presence of phages. *Bacillus amyloliquefaciens*‐mediated pathogen density reduction was clearest in the combinatory treatment. This suggests that simple laboratory experiments can qualitatively predict microbial interactions in much more complex rhizosphere. One crucial difference between these study systems was that the level of phage resistance evolved considerably lower in the rhizosphere. This could have been due to spatial heterogeneity of the soil, which could have lowered bacteria–phage encounter rates and strength of selection, or alternatively, due to the presence of natural microbial competitors that could have constrained pathogen and phage population densities (Gomez and Buckling 2011; Gomez et al. 2015). Despite the difference in the level of resistance, phage selection still increased the pathogen susceptibility to ancestral *B. amyloliquefaciens* antibiotics. This result is in line with recent study showing clear PAS without clear changes in pathogen phage resistance evolution (Torres‐Barcelo et al. 2016). Alternatively, it is possible that pathogen and phage showed fluctuating coevolutionary dynamics in the soil and potentially lost their resistance to ancestral phage (Gomez and Buckling 2011). Pathogen could have thus evolved different levels of resistance to their contemporary phages in different treatments but we missed this because the resistance of evolved pathogens was measured only against the ancestral phage. More work is thus needed to better understand how phage selection affects the susceptibility to *B. amyloliquefaciens* antibiotics. Despite the lack of exact mechanism, our results suggest that even low levels of phage resistance can impose strong evolutionary trade‐offs for pathogen survival in the rhizosphere.

The combinatory treatment led to around 40% reduction in bacterial wilt disease incidence during the greenhouse experiment. It has been found previously that *B. amyloliquefaciens* T‐5 alone can confer up to 65% disease control efficiency depending on the crop season and the environmental temperature (Wei et al. 2015a). In our experiment, the *B. amyloliquefaciens*‐mediated biocontrol had a relatively smaller effect on its own. One reason for this difference could be that in contrast to previous studies we did not use organic fertilizer as a carrier for the *B. amyloliquefaciens*, but instead, inoculated the biocontrol agents directly to the rhizosphere soil. This suggests that *B. amyloliquefaciens* biocontrol efficiency could be also shaped by organic nutrients provided by organic fertilizers (Wei et al. 2011). The lack of phage effect could have been due to low phage survival in the rhizosphere (Gomez et al. 2015). Determining, and potentially improving, the phage survival in the rhizosphere will be studied in the future experiments.

In conclusion, here we show that evolutionary dynamics are important for regulating pathogen densities and subsequent disease dynamics in the plant microbiome: while selection by a phage led to rapid increase in phage resistance, this made the resistant pathogen genotypes more susceptible to the antibiotics produced by *B. amyloliquefaciens* T‐5, and lowered pathogen competitive ability in terms of reduced growth. Together these results suggest that evolution of species interactions depends on the community context and that pathogen biocontrol could be potentially improved by using multiple biocontrol agents that show synergistic effects at ecological or evolutionary level. In more general perspective, our results suggest that host–parasite interactions are sensitive to competitive interactions, and that rapid evolutionary dynamics are likely to be important for shaping disease dynamics in natural environments.

Associate Editor: B. Koskella

Handling Editor: P. Tiffin

## Supporting information


**Figure S1**. (A) The phage plaque morphology on bacterial overlay plates after 24 h of incubation at 30°C.
