# Supplementary material for: Parasites and competitors suppress bacterial pathogen synergistically due to evolutionary trade‐offs
Source: Evolution. 2016 Dec 27;71(3):733–46. doi: 10.1111/evo.13143 (PMC5347860; doi:10.1111/evo.13143)
Supplement: Supplementary file 1 — Figure S1. (A) The phage plaque morphology on bacterial overlay plates after 24 h of incubation at 30°C. [file EVO-71-733-s001.docx]

**Supporting Information**


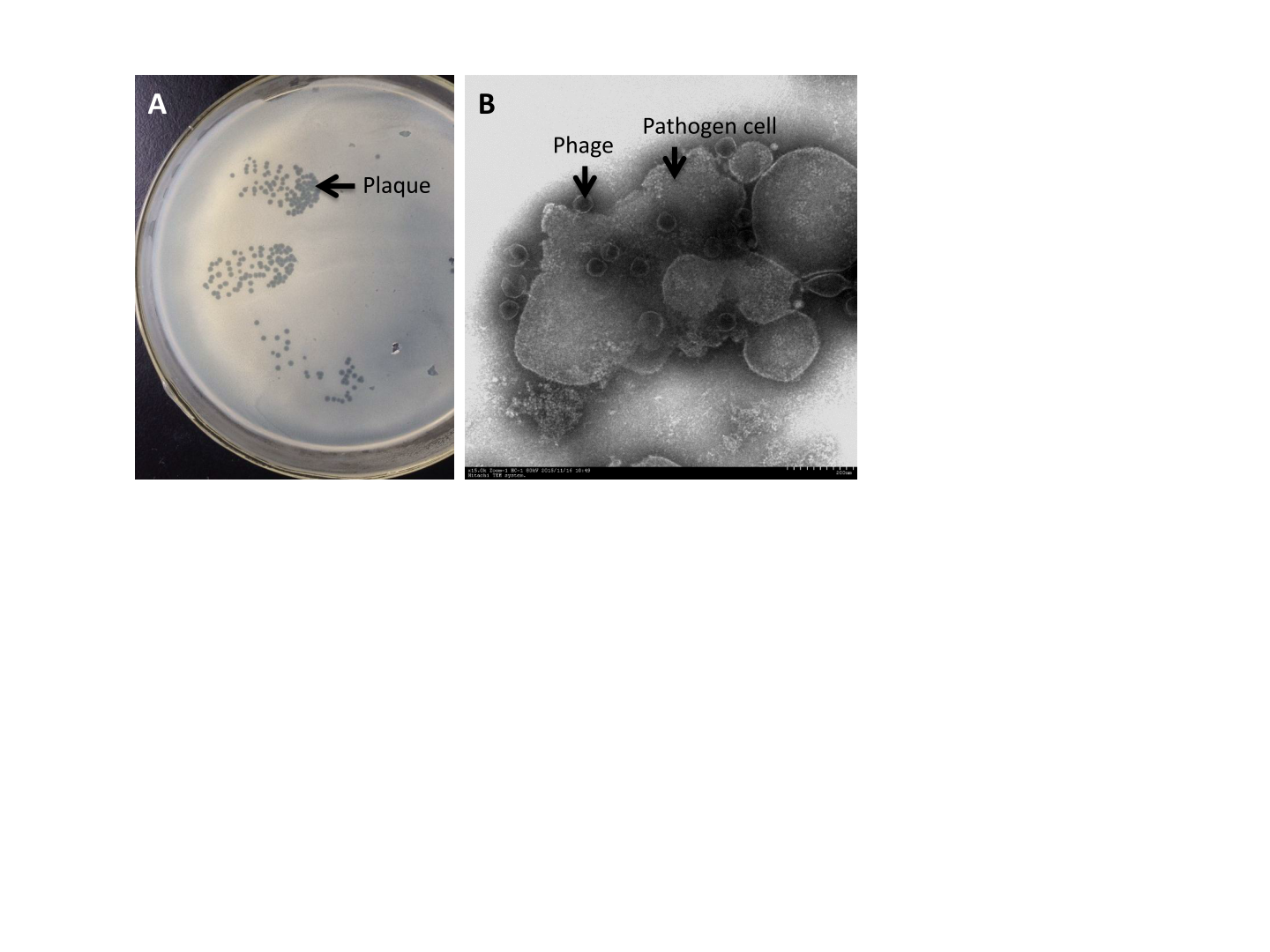


**Supplementary figure 1.** Panel (A): The phage plaque morphology on bacterial overlay plates after 24 h of incubation at 30°C. Panel (B): The phage morphology visualised with Transmission Electron Microscopy; phage particles were negatively stained with phosphotungstate. Bar = 200 nm.
